# Survival, Growth and Reproduction of Cryopreserved Larvae from a Marine Invertebrate, the Pacific Oyster (*Crassostrea gigas*)

**DOI:** 10.1371/journal.pone.0093486

**Published:** 2014-04-02

**Authors:** Marc Suquet, Catherine Labbé, Sophie Puyo, Christian Mingant, Benjamin Quittet, Myrina Boulais, Isabelle Queau, Dominique Ratiskol, Blandine Diss, Pierrick Haffray

**Affiliations:** 1 Ifremer, UMR 6539, PFOM Department, Station Expérimentale d'Argenton, Argenton, France; 2 INRA, UR 1037, LPGP, Rennes, France; 3 SYSAAF, LPGP, Rennes, France; 4 Satmar, Barfleur, France; University of New South Wales, Australia

## Abstract

This study is the first demonstration of successful post-thawing development to reproduction stage of diploid cryopreserved larvae in an aquatic invertebrate. Survival, growth and reproductive performances were studied in juvenile and adult Pacific oysters grown from cryopreserved embryos. Cryopreservation was performed at three early stages: trochophore (13±2 hours post fertilization: hpf), early D-larvae (24±2 hpf) and late D-larvae (43±2 hpf). From the beginning (88 days) at the end of the ongrowing phase (195 days), no mortality was recorded and mean body weights did not differ between the thawed oysters and the control. At the end of the growing-out phase (982 days), survival of the oysters cryopreserved at 13±2 hpf and at 43±2 hpf was significantly higher (P<0.001) than those of the control (non cryopreserved larvae). Only the batches cryopreserved at 24±2 hpf showed lower survival than the control. Reproductive integrity of the mature oysters, formely cryopreserved at 13±2 hpf and 24±2 hpf, was estimated by the sperm movement and the larval development of their offspring in 13 crosses gamete pools (five males and five females in each pool). In all but two crosses out of 13 tested (P<0.001), development rates of the offspring were not significantly different between frozen and unfrozen parents. In all, the growth and reproductive performances of oysters formerly cryopreserved at larval stages are close to those of controls. Furthermore, these performances did not differ between the three initial larval stages of cryopreservation. The utility of larvae cryopreservation is discussed and compared with the cryopreservation of gametes as a technique for selection programs and shellfish cryobanking.

## Introduction

Shellfish farming worldwide is mostly based on wild spat. The development of hatcheries combined with the recent adoption of genetic technologies such as selection or polyploidization (see for review [1], [2]) has created new genotypes with higher genetic value than wild stocks. The mid- and long-term cryopreservation of these new genetic resources has become of prime interest for research, genetic improvement (selection, hybridization or polyploidization) and for restoration programs for endangered species or populations. Obtaining optimized rearing potential for thawed embryos or larvae would result in an extension of the type of material that can be preserved in cryobanks [3].

Since the pioneering work carried out by Renard [4], most publications on the cryopreservation of shellfish embryos or larvae have been focused on the definition of protocols (cooling rate, type and concentration of cryoprotectant, embryo concentration in straws, etc…) and they have rarely reported good survival of thawed individuals or good performances after their settlement. In blue mussel (*Mytilus galloprovincialis*), the survival of 21-day old larvae after thawing was 12.5% of the control group [5]. Only 2.8% of the frozen/thawed Greenshell mussel (*Perna canaliculus*) trochophores survived up to competent pediveligers and their size was lower than that observed on unfrozen larvae [6].

In Pacific oysters, only one thawed larva succeeded in settling after 29 days of rearing [7]. During the growing-out phase, the survival of 850 four month old Eastern oyster (*Crassostrea virginica*), produced from thawed trochophore larvae, was not different from the control group [8]. However, this work did not provide information on later stages of development that could have validated larval cryopreservation as an effective tool for the maintenance of genetic resources. The recent improvement in survival reported from Pacific oyster larvae frozen after the trochophore stage [9] provided an opportunity to compare performances of one full generation in order to assess the feasibility of larval cryopreservation for genetic resources management purposes.

The aim of the present work was to describe the long-term effects of cryopreservation on the subsequent performances of Pacific oysters. The survival and growth rate of oysters which were cryopreserved at larval stages were assessed during the ongrowing and growing-out phases. Furthermore, the reproductive performances of these oysters were estimated up to the D-larval stage of their progenies.

## Materials and Methods

### Obtaining thawed larvae

In June 2009, oyster embryos were obtained from crosses made at the Ifremer experimental hatchery in Argenton (Northern Brittany, France; no specific permissions were required): three females were stripped during the natural spawning period. For each female 500 000 oocytes were fertilized in 300 ml seawater with a pool of sperm collected from three males (1.3 10^6^ spermatozoa ml^−1^) [10]. For each female, the embryos were incubated in 5L beakers (30 000 embryos L^−1^, 20°C). They were then pooled and batches were cryopreserved at 13±2 hours post fertilization: hpf (trochophore stage), 24±2 hpf (early D-larval stage) or 43±2 hpf (late D-larval stage), according to a protocol previously published [11] modified as follows: briefly, the larvae at the required stage were filtered on 20 μm mesh and diluted at a 1∶1 volume ratio in cryoprotectant (10% ethylene glycol with 1% PVP and 200 mM trehalose in bi-distilled water, final concentration). The larvae were frozen in 0.5 ml straws (23 000 larvae per straw, 18 straws per larval stage), using a Kryo 10 (Planer, Sunbury, U.K.) The freezing curve was: −1°C min^−1^ from 0 to −10°C, hold for 5 min at −10°C and then, −0.3°C min^−1^ from −10 to −35°C. Finally, the straws were plunged into liquid nitrogen. In September 2009, three months after cryopreservation, the straws were thawed in a water bath (37°C, 10 s) and the larvae were reared in a 5 L flow through culture system for 19 days (24°C), according to a method previously developed by Rico-Villa et al. [12]. A control group was created using a pool of oocytes and spermatozoa collected from three females and three males. At the end of the larval rearing phase which lasted 21 days, the survival was: 28.9±25.8% in the fresh control, 0.1±0.0% in the 13±2 hpf batch, 0.9±0.7% in the 24±2 hpf batch and 0.4±0.1% in the 43±2 hpf batch. Spat (juvenile oysters) were then filtered on 180 μm mesh, transferred to mesh bottomed sieve tanks (45×20×6 cm, mesh size: 150 μm) and maintained in a race way (flow rate: 12 L h^−1^ tank^−1^, seawater 24°C). They were fed daily with a mixture of two micro-algae (*Isochrysis galbana* and *Chaetoceros calcitrans*: at 65% and 35% respectively, maintaining a volume of 1 500 μm^3^ algae μl^−1^ seawater) for a 2 month period (November-December 2009).

### Ongrowing phase (this phase refers to 1 to 4 g oysters and aged of 88 to 195 days: the age is given without taking into account the period during which larvae were maintained in liquid nitrogen)

After filtration on a 4 mm mesh, spat were maintained in the same mesh bottom sieve tanks and reared according to the conditions described above. Spat number in each tank and the number of tanks were adapted to the number of spat available in each group, this number being related to the initial spat survival for each treatment (control: 280 spat/3 tanks; oysters produced from larvae cryopreserved at 13±2 hpf: 110 spat/1 tank, 24±2 hpf: 290 spat/3 tanks and 43±2 hpf: 245 spat/1 tank). Spat were counted and weighed individually (n = 30 per batch) at the end of the 4-month period (January to April 2010). The survival was calculated from the number of spat at the beginning of this ongrowing phase.

### Growing-out phase (4–80 g, 196–982 days)

The spat were then transferred to the natural site of Aber Benoit (North Brittany, France) where water temperature ranges from 9 to 17°C (The owner of the site gave permissions to conduct the field study and this study did not involve endangered or protected species). Spat were maintained in oyster bags (90×45×6 cm, mesh size 1.5 cm), spat number and number of bags were the same as those of the tanks in the ongrowing-phase. Oyster survival and mean weight (n = 30 per batch) were assessed at the end of the 26 month rearing period (April 2010-June 2012).

### Reproduction phase (>80 g, >982 days)

In June 2012, adult oysters were transferred from the Aber Benoit rearing site to the Ifremer experimental hatchery in Argenton, during the natural spawning period and their reproductive performances were assessed. Five groups of parents were created using five males and five females in each case: one group was the control made with the oysters which had never been frozen, two groups were composed of oysters formerly cryopreserved at 13±2 hpf (13.1 and 13.2) and the two last groups contained oysters produced from larvae cryopreserved at 24±2 hpf (24.1 and 24.2).

Gametes from each group were individually collected and pooled according to previously published techniques [10]. Therefore, each group produced one pool of sperm (from the five males in the group), and one pool of eggs (from the five females in the group). Sperm movement characteristics were assessed for each of the five pools of five males (13.1, 13.2, 24.1, 24.2 and control). Sperm motility was estimated using a two-step dilution procedure: firstly, 10 μL sperm (sperm concentration: 10^9^ to 10^10^ sperm ml^−1^) were diluted in 90 μL SW (seawater, salinity 35‰ +5 g L^−1^ BSA, 19°C), secondly, 2 μL of this first suspension was diluted again in 100 μL SW (final dilution 1∶500). Sperm samples of 7 μL were transferred to a Thomas cell and the movement characteristics were observed under a phase contrast microscope (Olympus BX51, ×20 magnification), connected to a camera (Qicam Fast 1394). The percentage of motile spermatozoa and their VAP (Velocity of the Average Path) were assessed using a CASA plug-in developed for the Image J software [13]. Calibration settings of the software were as follows, frame rate: 25 frame sec^−1^; sperm size range: 0.9 to 7.5 μm; minimum VAP for motile sperm: 10 μm sec^−1^; minimum track length: 6 frames; minimum number of sperm observed: 30.

The fertilization capacity of the four sperm pools (13.1, 13.2, 24.1, 24.2) was then estimated in triplicate (50 000 oocytes for each pool; fertilization volume 100 ml) [10]. Firstly, each sperm pool was used to fertilize a pool of oocytes collected from the control group (four crosses). Secondly, the sperm was used to fertilize the oocytes of its respective female groups (four crosses). Because sperm quality was being studied, a limiting sperm to oocyte ratio of 300, corresponding to 150 000 sperm ml^−1^, was used. Furthermore, oocyte quality was estimated using the four oocyte pools (13.1, 13.2, 24.1, 24.2) and a pool of fresh sperm collected from the control group (four crosses). The pools of oocytes were fertilized using the method described above (except that a non limiting sperm to oocyte ratio of 800 was used to study oocyte quality, corresponding to 400 000 spermatoza ml^−1^). Then, a control (male control x female control) was added. For both sperm and oocyte quality studies, the number of D-larvae was counted 48 hours post fertilization (3×100 μL samples) for the 13 crosses carried out. The D-larval survival was calculated as a percentage of the oocyte number used for fertilization.

### Statistical analysis

Data are presented as mean ± standard deviation. Percentages were arcsin square root transformed before analysis. Means were compared using one way ANOVA. When differences were significant (P<0.05), a Tukey a posteriori test was used for mean comparison.

## Results

**Figure 1:**
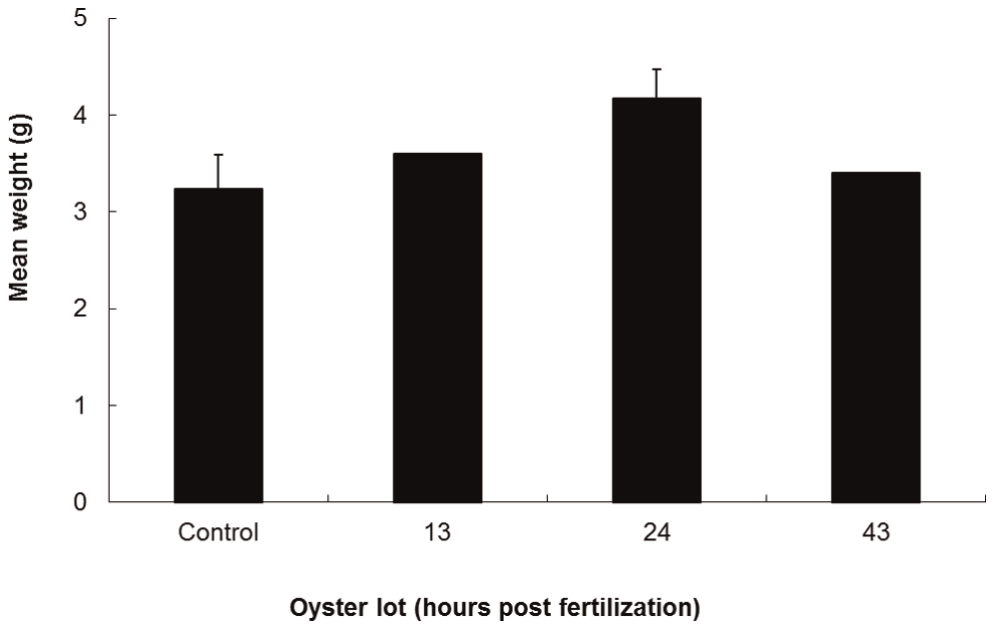
Effect of larvae cryopreservation at different stages post fertilization on the subsequent growth rate of oysters at the end of the ongrowing phase (195 days; spat number in each tank/number of tanks, control: 280/3, 13±2 hpf batch: 110/1, 24±2 hpf batch: 290/3, 43±2 hpf batch: 245/1).

At the end of the ongrowing phase, no mortality was recorded in the four different oyster batches (control, oysters produced from larvae cryopreserved at 13±2 hpf, 24±2 hpf and 43±2 hpf). Furthermore, the mean weights of the four oyster batches were not significantly different (Figure 1).

**Figure 2:**
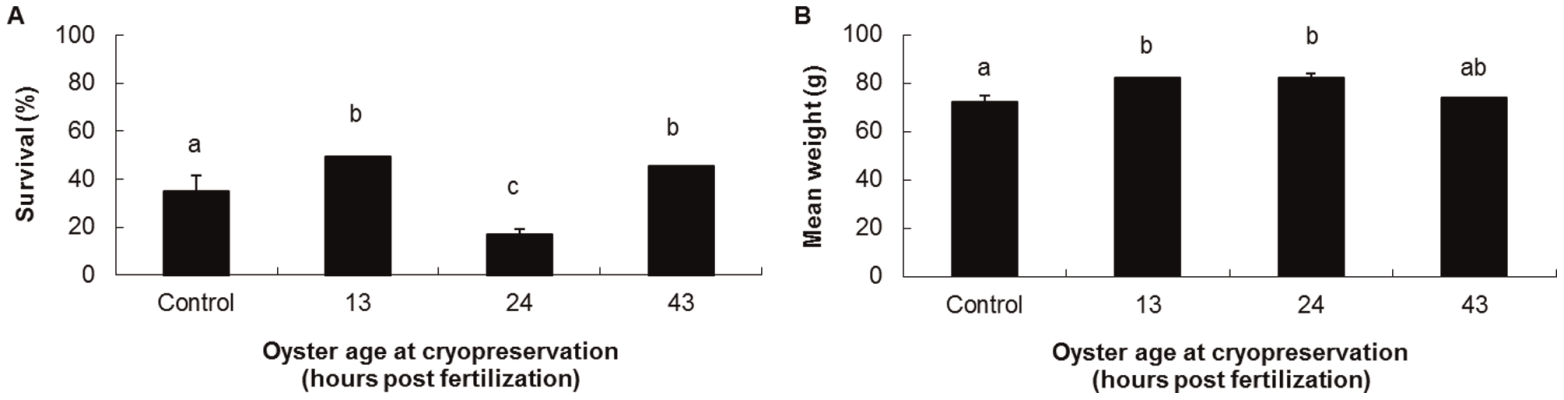
Effect of larvae cryopreservation at different stages post fertilization on the subsequent performances of oysters at the end of the growing-out phase (982 days). A: survival, B: mean weight observed at the end of the phase (spat number in each bag and number of bags are the same as those reported for Figure 1; different letters indicate significantly different results).

At the end of the growing-out phase, the survival of two cryopreserved batches (13±2 hpf and 43±2 hpf) was significantly higher than the control (Figure 2). Only the 24±2 hpf had survival lower than the fresh control (P<0.001). The mean weight of the cryopreserved batches was either higher (13±2 hpf and 24±2 hpf; P<0.05) or similar (43±2 hpf) to that of the control.

**Figure 3:**
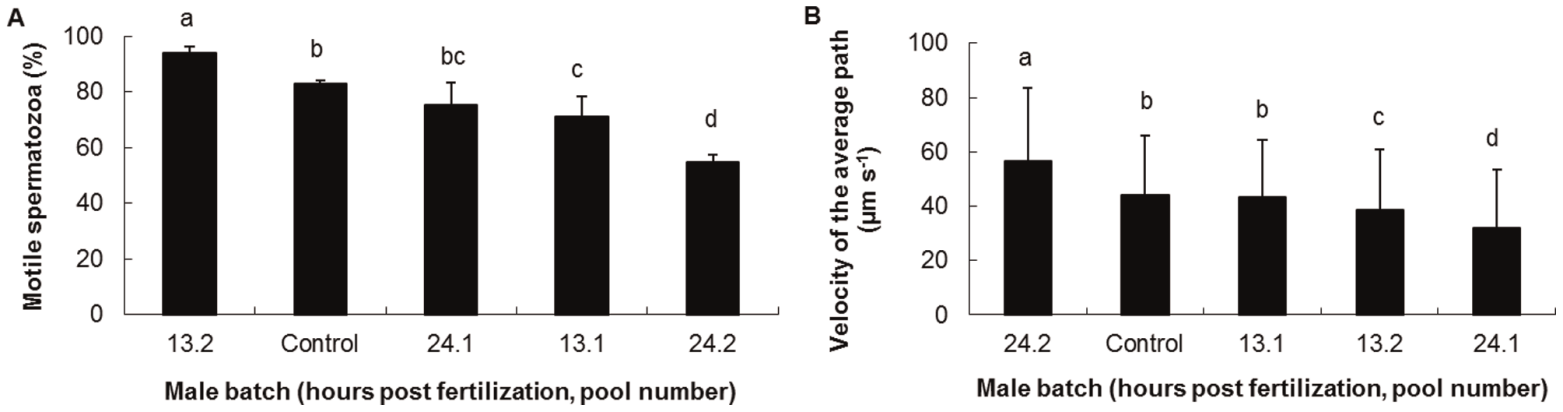
Effect of larvae cryopreservation at different stages post fertilization on the subsequent performances of oysters during the reproductive phase (>982 days). A: percentage of motile spermatozoa, B: Velocity of the Average Path (n>30 spermatozoa scored for each male gamete pool; n = 5 males contributed to each pool; different letters indicate significantly different results).

During the reproductive phase, the percentage of motile spermatozoa observed for pool 13.2 (cryopreservation 13±2 hpf of the gamete pool n°2) was significantly higher (P<0.001) than that observed for the four other pools (control, 13.1, 24.1 and 24.2; Figure 3). A percentage of motile spermatozoa higher than 70% was recorded for four of the pools, but not for pool 24.2. This observation highlights the good quality of the sperm produced in four pools out of five. The VAP observed for the pool 24.2 was significantly higher (P<0.001) than that of the control. Furthermore, the VAP observed for the pools 13.2 and 24.1 was lower than that recorded for the control. In all, the VAP recorded for three (24.2, 13.1 and 13.2) out of the four sperm pools from thawed oysters was never below 70% of the VAP value recorded for the control.

**Figure 4:**
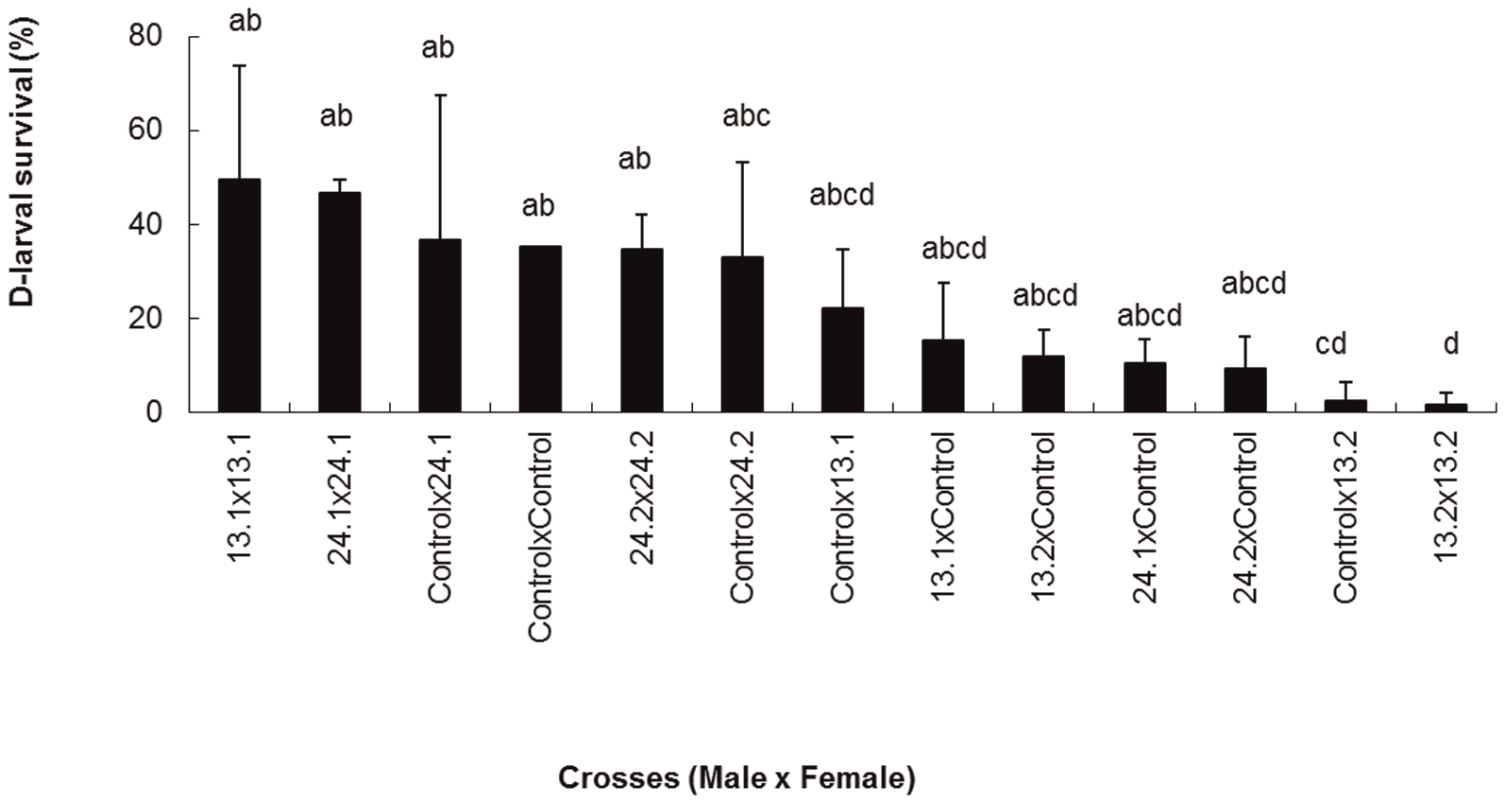
Effect of larvae cryopreservation at different stages post fertilization on the subsequent D-larval survival in progenies. (n = 5 oysters contributed to each gamete pool; different letters indicate to significantly different results).

Larges variations in the D-larval survival (1.5 to 49.6%; Figure 4) were observed depending on the crossing but they were not significantly different from the control (male control x female control), except for the progenies issued from control x 13.2 and 13.2×13.2 (P<0.001).

## Discussion

This study reports survival and growth to reproduction at 2.7 years of age of thawed Pacific oyster larvae previously cryopreserved at three different larval stages. This is the first report of the successful development of cryopreserved individuals to reproduction in molluscs and the second report of viable progenies produced from cryopreserved parents in aquatic invertebrates. Indeed a study was already published on the rotifer, *Brachionus plicatilis* [14]. In this species, the thawed larvae started to lay eggs 2 to 3 days after thawing. Therefore, any damage caused by cryopreservation may not have had time to be expressed compared with oysters in the present work, as these reproduced more than 2 years after. Moreover, gamete quality was not evaluated in *B. plicatilis* because it reproduces by parthenogenesis, thereby producing amictic eggs. Similar positive results had already been reported in other invertebrates such as in insects (see review in [15]). However, major differences exist between such studies and the present model: permeabilization of the insect egg shell, deshydratation and vitrification are not required in Pacific oyster. The simpler cryopreservation procedure used in oyster is associated with the eco-physiology of marine mollusc larvae which do not have a thick external cuticle that could limit osmotic exchanges and cryoprotectant efficacy.

In the present study, no marked differences were observed in the ongrowing, growing-out and reproductive capacities of oysters produced from cryopreserved larvae compared with those of oysters that had been grown directly from fresh larvae with no cryopreservation step. These results confirm a pioneering study carried out by Paniagua-Chavez and co-workers [8], assessing the effects of cryopreservation at the trochophore stage on the subsequent development of Eastern oysters until the age of four months after thawing: the survival of larvae produced from thawed embryos was not different from the control. Furthermore, our work compares the effect of cryopreservation at three different stages of development. No marked differences were found in the rearing performances between these treatments and the control group. This indicates that after the initial dramatic mortalities observed during the larval development, the surviving larvae yielded similar post-settlement rearing performances to the ones that have never been frozen. The protocol of the present study differs from that used by Paniagua-Chavez et al. [8] in many respects (equilibration phase, cooling rate, cryoprotectant and thawing temperature). Because of similar conclusions at least for the immature stage, it can be suggested that there is only a limited interaction between the freezing protocol and the performances of the thawed larvae after their settlement. However, this hypothesis must be confirmed after the improvement of initial larvae survival observed just after thawing.

Some limitations of the study include the long-term protocol used. Firstly, oyster density maintained in tanks during the ongrowing phase and in bags during the growing-out phase differed between batches. Oyster density of each batch depended on the survival observed during the previous rearing phase. However, these densities are considered as non limiting for oyster growth [16]. Furthermore at the end of the ongrowing phase, the low survival observed for the 24±2 hpf batch which had the highest oyster density was moderated by its high growth rate assessed during the same rearing phase and its high subsequent reproductive performances. Secondly, parental effects on rearing performances of spat produced from thawed larvae were not studied in the present work. Such effects could influence a number of different rearing parameters: the survival of D-larvae produced from thawed oocytes collected from eight Pacific oyster females ranged from 0.1 to 30.1% [11]. However, the present 32 month protocol would be too difficult to conduct because it would require keeping separated progenies from different families. As a consequence, a pool of females and a pool of males were used to obtain the larvae used for cryopreservation. This choice is similar to previous studies aiming to describe the long-term rearing performances of molluscs produced from thawed larvae [5], [6].

The high rearing performances of surviving thawed larvae may suggest an absence of genome alterations on the individuals that succeeded in settling, allowing subsequent development of these larvae. DNA alteration of thawed embryos has mostly been investigated in mammalian species [17] but rarely in invertebrates. Several instances of DNA damage were identified in the ragworm (*Nereis virens*) [18] and in insects [15], the most life-threatening of these is thought to be damages to the gut epithelium in ragworm. DNA integrity has been more greatly studied in cryopreserved fish gametes and several techniques to assess DNA integrity have been developed, including Tunel (Terminal deoxynucleotidyl transferase-nick-end-labelling), SCSA (Sperm Chromatin Structure Assay) and Comet assays (single gell electrophoresis) [19]. Inter-species differences have been found: cryopreservation caused damage to rainbow trout (*Onchorhynchus mykiss*) sperm compared with fresh sperm, but not to sperm of gilthead sea bream (*Sparus aurata*) [20]. In molluscs, the percentage of Pacific oyster spermatozoa with damaged DNA significantly increased after cryopreservation [21] but the consequences of such damage for the F_1_ progenies were not estimated. Because a principal application of cryopreservation is to maintain the genetic variability of domesticated and wild animal species, the long-term development capacities of thawed larvae must be studied.

The discussion sections of scientific papers on embryo cryopreservation are often generally limited to scientific or technical questions, without addressing the relative benefits offered by larvae cryopreservation compared to gamete cryopreservation. It was shown that the development capacities of surviving D-larvae up to spat stage was not altered by oocyte cryopreservation [11]. Together with the success of sperm cryopreservation, and with the results showed in our study on larvae, it can be inferred that both haploid and diploid genomes of Pacific oyster can now be cryopreserved. The freezing of haploid spermatozoa or oocytes or of diploid spermatozoa collected from tetraploid males may be useful for controlled crosses using parents presenting desired characteristics, thus supporting the production of original genotypes for genetic selection and of triploids. The rationale of larvae cryopreservation is different since it cannot create new genetic variation *per se* but preserves diploid family genomes already created by controlled crosses. The cryopreservation of larvae has several other interests for shellfish aquaculture. First, it secures investments in selection programs as the families created can be conserved frozen. This could allow breeder to return to the best families of earlier-made crosses for selection purposes (with a loss of a one generation interval for sib selection). Second, it could allow a rapid restoration of genetic variation from previous generations if genetic drift or inbreeding depression are observed. Third, it could allow inter-generation estimation of genetic progress, reducing costs and risks associated with maintenance of live control lines that are never really equivalent to a true control because of genetic drift across generations, non-intentional selection or domestication. More generally, the cryopreservation of diploid progenies may also provide suitable means for diffusing genetic progress and genetic exchange of material worldwide in using only sanitary approved genotypes ready to be used as future broodstock. The application of a cryopreservation procedure developed for diploid larvae may also facilitate maintenance and genetic improvement of tetraploid oyster stocks by cryopreservation of tetraploid larvae [2].

## Conclusion

In the present work, We demonstrated that the subsequent growing-out and reproductive performances of surviving thawed oysters are similar to those observed for unfrozen ones. From thawing up to the end of the growing out phase (982 days), oyster survival observed was respectively 0.05% (13±2 hpf), 0.15% (24±2 hpf), 0.18% (43±2 hpf) and 10.06% (control). Because of the very high fecundity observed in Pacific oyster (10 to 50×10^6^ oocytes per female), larvae cryopreservation allows the production of 11,000 to 42,000 good quality adult oysters for each female, depending on oyster development stage at cryopreservation. These yields can be largely increased by the further optimisation of the cryopreservation protocols designed for oyster larvae.

Pacific oyster is the first aquatic farmed species for which sperm, oocyte and larval genomes can be stored in this way. Freezing larvae will help to preserve the genomes of wild oyster populations and of selected oyster families. Furthermore, thawed oysters may be used to evaluate the genetic progress recorded between successive generations. Such advances in embryo cryopreservation will contribute to the establishment of oyster cryobanks and maintenance of the genetic diversity of domestic and wild stocks of other mollusc species.
